# A new strategy based on pharmacophore-based virtual screening in adenosine deaminase inhibitors detection and in-vitro study

**DOI:** 10.1186/2008-2231-20-64

**Published:** 2012-10-22

**Authors:** Roya Bazl, Mohammad Reza Ganjali, Ali-akbar Saboury, Alireza Foroumadi, Parviz Nourozi, Massoud Amanlou

**Affiliations:** 1 Center of Excellence in Electrochemistry, Faculty of Chemistry, University of Tehran, Tehran, Iran; 2 Institute of Biochemistry and Biophysics, University of Tehran, Tehran, Iran; 3 Department of Medicinal Chemistry, Faculty of Pharmacy, Drug Design & Development Research Center, Tehran University of Medical Sciences, Tehran, Iran

**Keywords:** Adenosine deaminase, Pharmachophore, Docking, Lead discovery, Inhibitor

## Abstract

**Background and the purpose of the study:**

Adenosine deaminase (ADA) inhibition not only may be applied for the treatment of ischemic injury, hypertension, lymphomas and leukaemia, but also they have been considered as anti- inflammatory drugs. On the other hand according to literatures, ADA inhibitors without a nucleoside framework would improve pharmacokinetics and decrease toxicity. Hence we have carried out a rational pharmacophore design for non-nucleoside inhibitors filtration.

**Methods:**

A merged pharmacophore model based on the most potent non-nucleoside inhibitor erythro-9-(2-hydroxy-3-nonyl) adenine (EHNA) and natural products were generated and applied for compounds filtration. The effects of filtrated compounds based on pharmacophore and docking studies investigated on ADA by UV and Fluorescence spectroscopy techniques.

**Results:**

Extracted compounds were find efficiently inhibit ADA, and the most potent (**2**) shows an inhibition constant equal to 20 μM. Besides, Fluorescence spectroscopy studies revealed that enzyme 3D structure bear further change in lower concentrations of compound **2**.

**Conclusion:**

3 non-nucleoside inhibitors for ADA are presented. According to obtained results from UV and fluorescence spectroscopy, such interesting pharmacophore template with multiple approaches will help us to extract or design compound with desired properties.

## Introduction

Adenosine deaminase (ADA) is a key enzyme in the purine metabolism that hydrolyse adenosine to inosine irreversibly [1]. This path involves in RNA, DNA, ATP synthesizes, and energy transitions reactions. This enzyme also has been found in lymphoid systems like lymph nodes, spleen and thymus [2]. Involvement of this enzyme is clear in catabolytic paths and also its role in the protection of immune systems [3-5].

Over activity of ADA is associated with AIDS, leukemia, stresses and Parkinson [6-8]. In addition, the high value of ADA has been seen in rheumatoid arthritis [9]. Adenosine as the substrate of ADA regulates many of physiological processes in different organisms [10,11]. Adenosine influences deeply on hypertension, sedation and vessels dilatation [12]. Also, it acts as nerve modulators or as neural hormones [13].

On the other hand, most of adenosine analogues have more importance in chemotherapy, cancer, immunology, virology and parasitology, which could be deaminated by the enzyme and deactivated through their metabolic pathway [14]. So, inhibition of ADA can solve mentioned problems [15,16]. Till now, suggested inhibitors have some drawbacks such as irreversibility, side effects, high inhibition constant (K_i_) and toxicity on the different cells [17]. Moreover, most of existing nucleoside inhibitors not only have difficulties in their synthesis, but also because of interfering with function of other enzymes they have been deleted from researches pathways despite of appropriate inhibition potency [18].

Natural sources are receiving increasing attention recent years since they were reported to have a remarkable spectrum of biological activities including antioxidant, anti-inflammatory and anti- carcinogenic activities [19-21]. On the other hand, several methods have been raised in drug discovery such as high throughput screening, docking and QSAR analysis [22]. The designed compounds were more investigated based on sequential filters and at last selected compounds were more studied in biological tests.

Since experimental methods are time consuming, computational techniques such as docking and virtual screening (VS), help researchers to gain effective compounds in shorter time and lower costs [23]. The aim of this project is to develop merged Pharmachophore model based on the most potent non-nucleoside inhibitor EHNA and natural products from ZINC data base compounds which have effective interaction with active site of enzyme. This model was applied for filtration of effective inhibitors for ADA from the in-house data base, and their efficiencies are determined through biological investigations.

### Experimental

#### Chemicals

Adenosine deaminase (from bovine spleen in 3.2 M ammonium sulfate) was purchased from Sigma (St. Louis, MO, USA). Phosphate buffer 50 mM, pH 7.5, was used as media which is comprised NaH_2_PO_4_ and Na_2_HPO_4_ and are from Merck. In addition, other material such as solvents, were purchased from this company.

#### Virtual screening

Crystal structure 1KRM from bovine for ADA was extracted as raw enzyme structure with 80% identity to applied enzyme in biological tests. Autogride4.2 and Autodock4.2 were used for calculation of grid maps and docking, respectively. AutoDockTools 1.5.4 was used for preparing input files. In this study the compounds were docked on ADA with the grid-box of 126 Å (x, y and z) with the spacing of 0.375 Å. Docking calculation parameters were set to these values: number of Lamarckian job =100; initial population =100; maximum number of energy evaluations =25×10^5^; maximum generations =27000; mutation rate of 0.02; a crossover rate of 0.80. For better understanding of interactions and access to respective pharmacophore, LIGSCOT3.0 program was used.

#### Biological tests

Biological tests have been done on synthesized compounds from Tehran University of Medical Sciences in-house library. All of biological samples were prepared with double distilled water. The adenosine stock solution prepared and was used in concentration range of 0.01-0.4 mM. The concentration of enzyme in the assay mixture 50 mM sodium phosphate buffer, was 0.9 nM with a final volume of 1ml. Enzymatic activities were assayed by UV–vis spectrophotometry (Shimadzu-3100, Japan) based on Kaplan Method to follow the decrease in absorbance at 265 nm resulting from the conversion of adenosine to inosine [24]. Determination of enzyme activity in the presence of different concentration of substrate was showed that the increasing concentration of substrate from 10 to 124 μM enhanced the enzyme activity. But this activity reached to zero in the higher concentration of substrate (more than 400 μM). This effect may be due to suicide-like mechanism in higher concentration of adenosine.

Fluorescence study has been done with fluorescent spectroscopy (Hitachi MPF-4, Japan), which its cell length is 1cm. In this method, the exciting wavelength was adjusted in 290 nm and the emission was studied in range of 300–400 nm for possible enzyme 3-D structure changes. In this experiment, different concentrations of inhibitors (5–100 μM) were investigated in the presence of ADA (0.01unit) by final volume of 400 μL with phosphate buffer.

## Results

### Validation

To validate the method in prediction of binding energy and possible interaction between ligands and receptor, at first existing ligand in crystal structure was re-docked in 1KRM. After superimposition RMSD <0.1 was gained and showed that this technique and applied parameters would be applicable for next predictions.

### Extraction of compounds from Tehran University of Medical Sciences database

**Figure 1:**
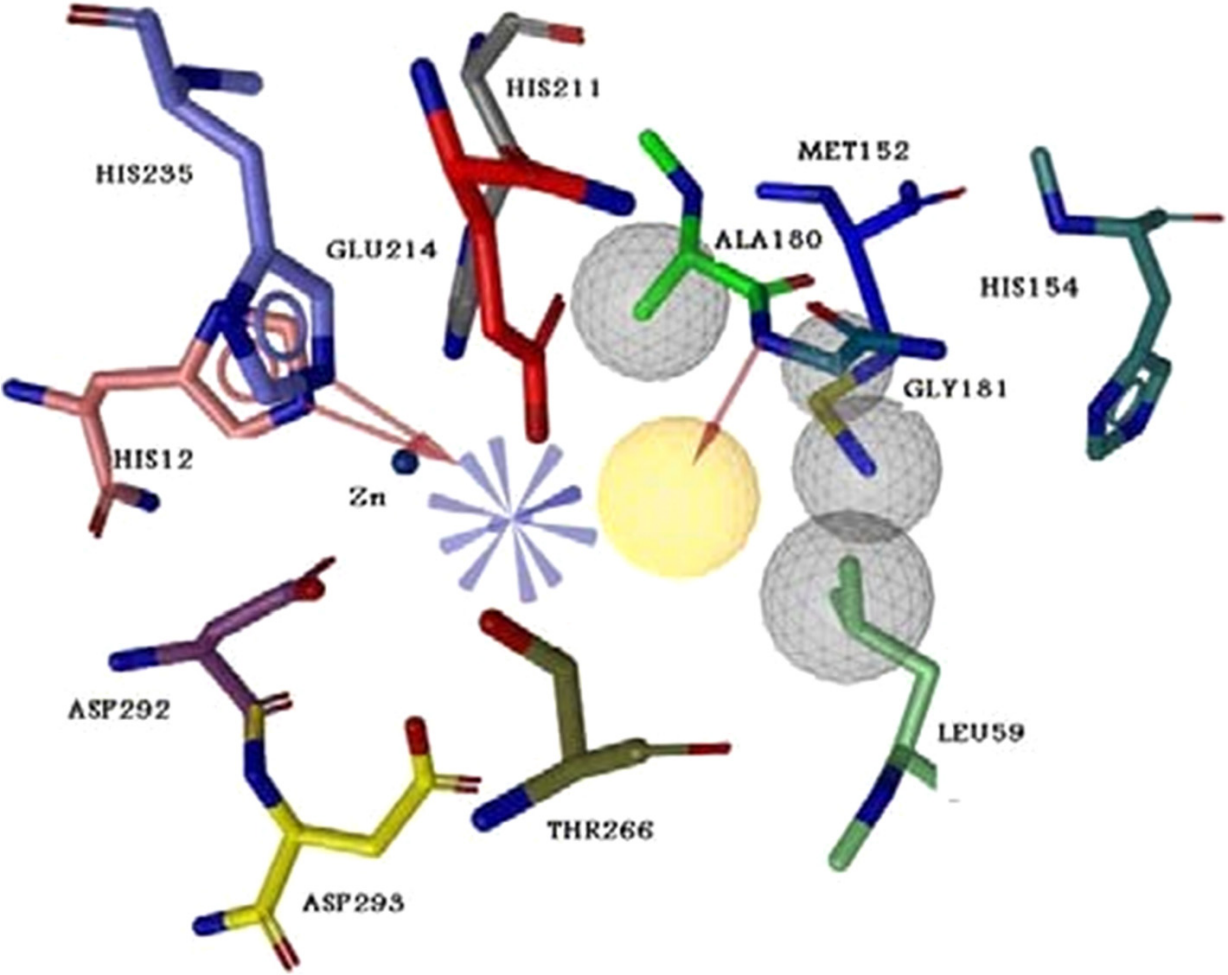
**Merged pharmacophore from most potent non-nucleoside inhibitor of adenosine deaminase (EHNA) and effective docked natural products from ZINC data base.** Yellow area are demonstrated as Hydrophobic area, gray cycle as exclusion volume regions, red dashes as H-bond acceptor part and blue dashes as positive region.

In first stage, 8000 natural compounds of ZINC database were filtered according to lead likeness and were prepared for docking on the ADA. 25% of achieving compounds were filtered with best energy and transmitted to next stage for docking with higher energy values. After that, the compounds with acceptable orientation in enzyme active site were filtered and binding energy estimation was performed in grid box dimension of 60×60×60 Å. The achieved compounds with best binding energy include imidazole rings and NO_2_ group in their position 2. As it demonstrated in Figure 1, the related pharmacophore was extracted with Ligandscout software and merged with extracted pharmacophore from non-nucleoside potent inhibitor, EHNA (K_i_=37 nM) [25].

**Figure 2:**
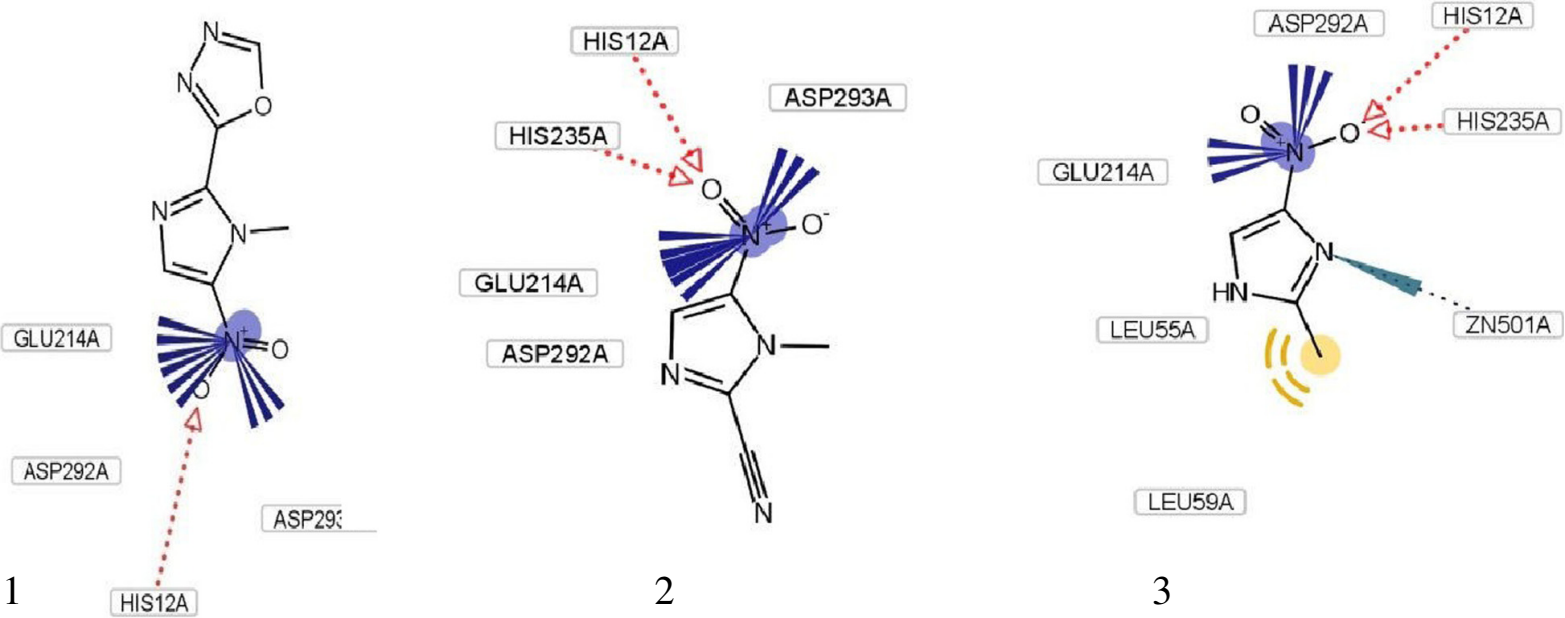
The proposed binding mode of three extracted compound from in-house library as determined by docking calculations within the catalytic site of adenosine deaminase.

In next stage, the structures of 540 synthesized compounds from in-house library have been sketched with Marvin, and were optimized by Hyperchem software. Then, compounds were filtered by designed merged pharmacophore and obtained compounds (15 compounds) were docked again and three compounds with proper fitting in active site and lower binding energies were extracted Figure 2.The related binding energies are summarized in Table 1.

**Table 1:** **Summary of binding energies, theoretical K**_**i**_**, IC**_**50 **_**and inhibition constants of new inhibitors for calf intestinal adenosine deaminase**

**Compounds**	Δ**G° (kcal/mol)**	**Calculated K**_**i**_**(μM)**	**IC**_**50**_**(μM)**	**K**_**i**_**(μM)**
1	−7.02	27	103	63
2	−9.59	0.1	41	20
3	−8.37	15	85	45

### Biological assay

**Figure 3:**
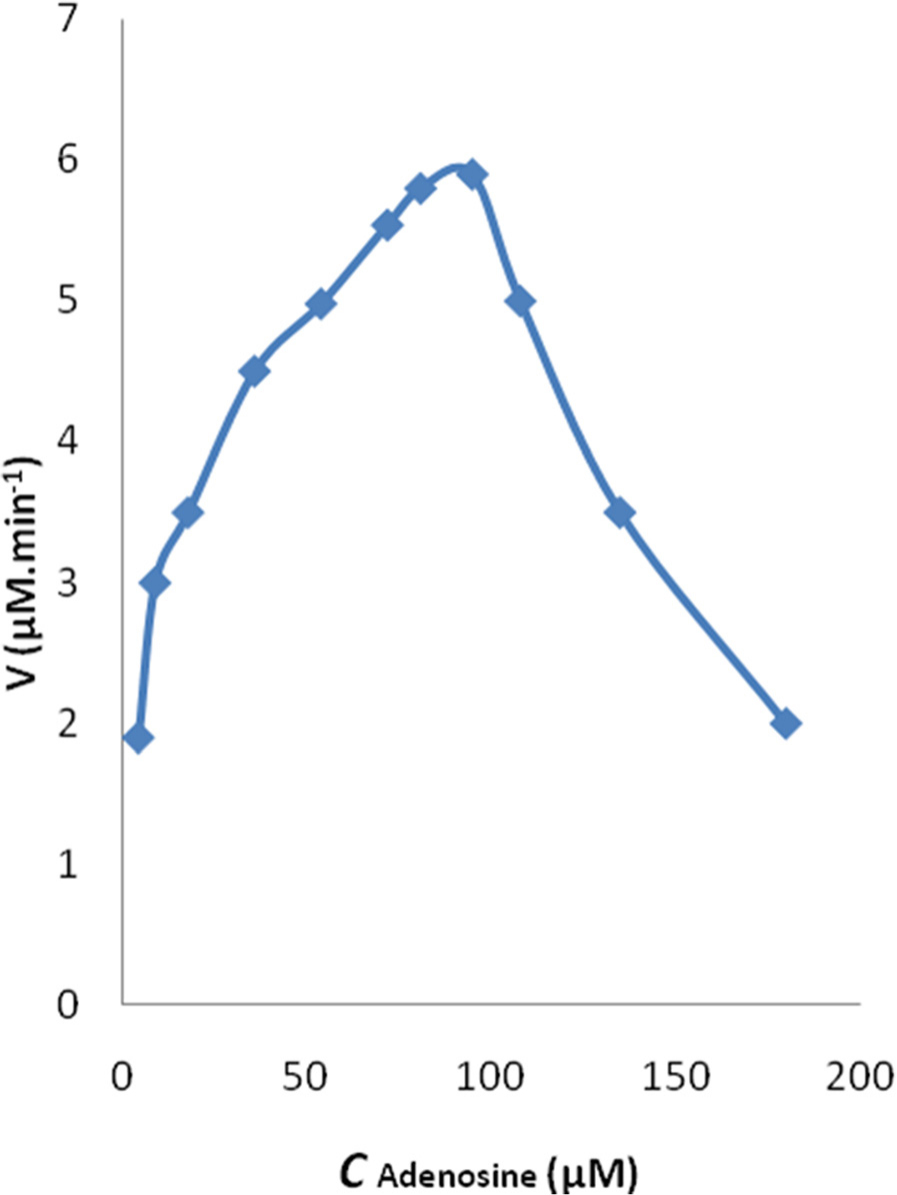
**A Michaelis–Menten plot was used to determine K**_**m **_**and V**_**max**_**. The calculated K**_**m **_**was 37.6 μM and V**_**max **_**was 15.7 μM min**^**-1 **^**(n = 3)**.

Figure 3 shows a Michaelis-Menten plot in the presence of different concentration of adenosine at pH=7.5 and 37°C. The enzyme shows the most activity in 124 μM of substrate with acceptable K_m_=37.6 μM.

For accurate determination of kinetic parameters, the IC_50_ values were evaluated, and then the enzyme inhibition was studied in 180 s intervals. The resulted IC_50_ values are listed in Table 1. For more inside through mechanism of inhibition, kinetic studies were performed and all three compounds show a competitive inhibition mechanism. In a competitive inhibition; V_max_ is unaltered, whereas K_m_ is increased. The slope of a competitive plot is equal to:

$$\text{Slope=}\;\frac{K_{m}\;\left[I\right]}{V_{\text{max}.}K_{i}}\;+\frac{K_{m}}{V_{\text{max}}}$$

**Figure 4:**
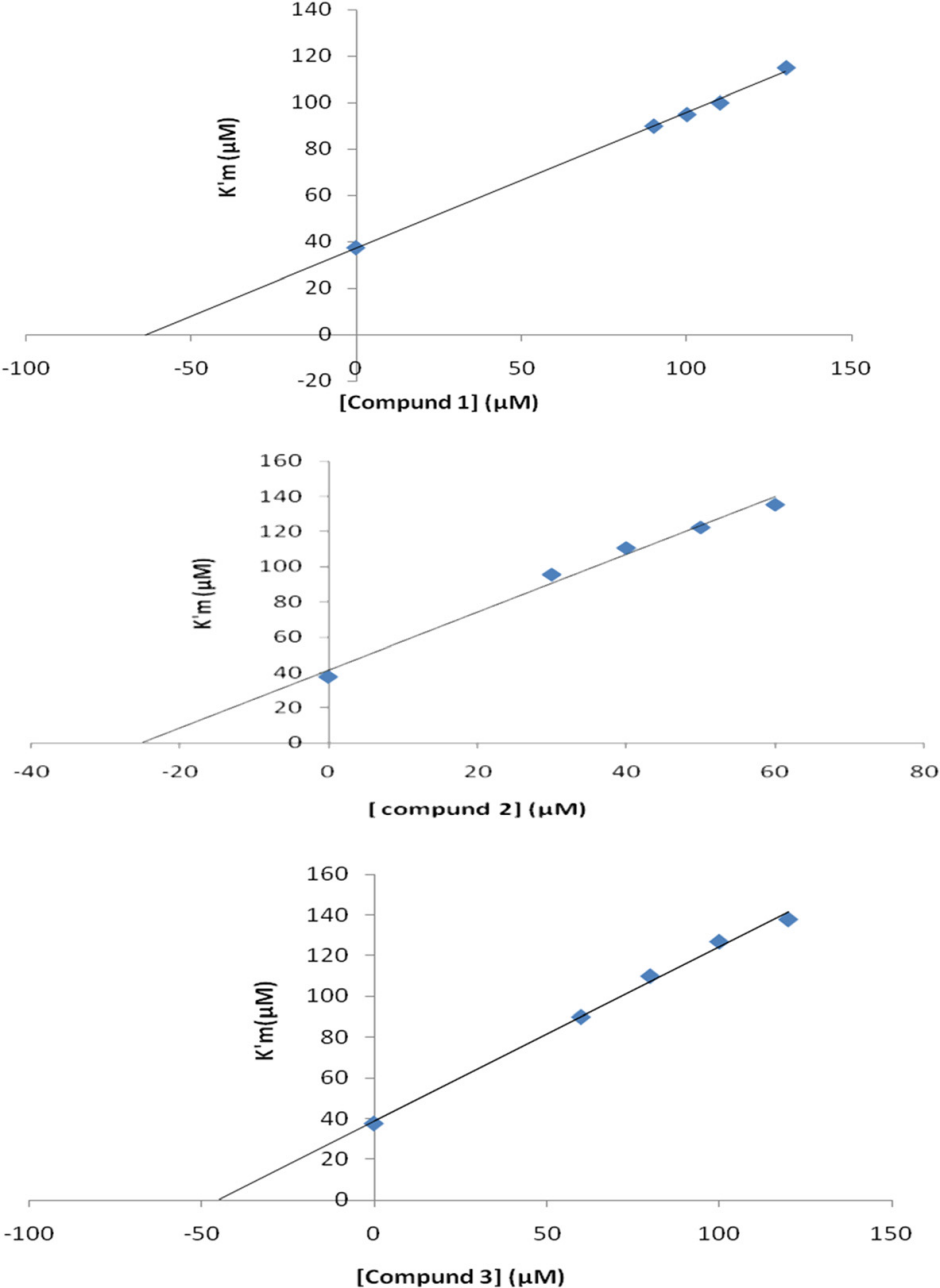
**Secondary plot, K**^**′**^**m versus three novel inhibitors in the range of 0–130 μM and in different concentration of substrate 20–124 μM.** All experiments performed in 37°C and phosphate buffer, pH=7.5.

A plot of apparent Km values versus concentration of inhibitors [I] results to the inhibition constants which is demonstrated in Figure 4.

**Figure 5:**
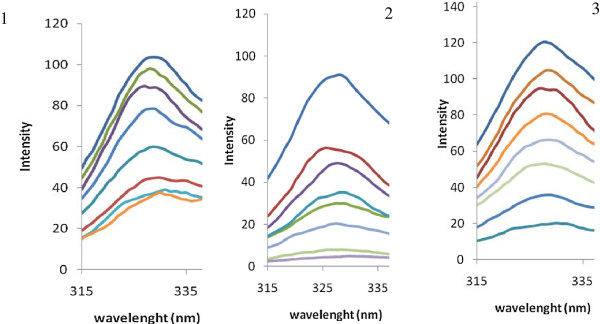
**The fluorescence spectra of adenosine deaminase at pH=7.5 and T=37°C in the presence of different concentrations of three inhibitors: 10–100 μM**.

To investigate changes in enzyme 3-D structure at different concentration of inhibitors (10–100 μM), the fluorescence experiments were done in excitation wavelength of 290 nm, which is due to tryptophan amino acids in the active site of enzyme Figure 5.

## Discussion

According to importance of enzyme inhibition in treatment of some diseases [26], in this research we use a new approach in better understanding and filtering of specific ADA inhibitors. After pharmacophore generation, docking was performed in order to rank the in-house compounds on the basis of their ability to form favorable interactions within the active site of ADA. This approach not only helps us to filter compounds according to their similarity to most potent non-nucleoside existing inhibitor, but also helps us to extract compounds with natural template. As it shown in Figure 2, imidazole derivative compounds properly stabilize in the binding pocket of ADA with different kinds of interaction such as, hydrophobic, electrostatic, H-bond and π-π stacking. Previously potent inhibitors with imidazole ring have been reported for ADA inhibition [27]. From Table 1, the calculated ΔG° energies are so low which shows effectiveness of compounds in inhibition. Indeed, resulted K_i_ from biological tests improves this subject. Among, compound **2** shows better interaction with lower binding energy with enzyme −9.59 kcal mol^-1^.

ADA is inhibited by three filtrated compounds through competitive mechanism, which is in agreement with docking studies. This mechanism can attribute to size of molecule which able compounds penetrate in enzyme active site and make appropriate interactions. Changing in temperature can affect on the reaction rate and enzyme activity. As can be seen in Table 1, resulted K_i_ from inhibition investigation was higher than expected from docking studies according to their binding energies. This is probably due to difference in temperature was used in biological test, 37°C which enzyme has maximum activity in, and docking approach which subjected to 25°C according to Gibbs free energy equation. However, calculated binding energies and experimental K_i_ values are following logical trend (r^2^=0.99). According to fluorescence intensities for three obtained inhibitors, ADA shows sever structural changes in lower concentration of **2** (10 μM). Florescence, kinetic study and appropriate interaction of compound **2** in enzyme active site have confirmed its potency as competitive ADA inhibitor.

## Conclusion

This study has showed the effective application of docking and pharmacophore studies in introducing new inhibitors for target receptor. Designed merged pharmacophore results in extraction of effective compounds from in-house 540 compounds, and subjected them to experimental phase for inhibition evaluations. The resulted three compounds from VS studies showed inhibitory effects through competitive mechanism on ADA. Compound **2** can be further optimized and used as a lead compound in new inhibitor design with more potency.

## Competing interests

There are no other conflicts of interest related to this publication.

## Authors’ contributions

All authors contributed to the concept and design, making and analysis of data, drafting, revising and final approval. MA is responsible for the study registration. RB & MA, AAS and MRG for conception and design, RB, AF, AAS and MA for provision of study material, RB, MA and AAS for collection and/or assembly of data, data analysis, interpretation and manuscript writing and RB, MA, MRG and AAS for financial and administrative support. All authors read and approved the final manuscript.
